# Prospective study of oral pre‐exposure prophylaxis initiation and adherence among young women in KwaZulu‐Natal, South Africa

**DOI:** 10.1002/jia2.25957

**Published:** 2022-07-03

**Authors:** Leila E. Mansoor, Lara Lewis, Cherise L. Naicker, Ishana Harkoo, Halima Dawood, Kalendri Naidoo, Tanuja N. Gengiah, Natasha Samsunder, Ivana Beesham, Salim S. Abdool Karim, Quarraisha Abdool Karim

**Affiliations:** ^1^ Centre for the AIDS Programme of Research in South Africa (CAPRISA) Nelson R Mandela School of Medicine, University of KwaZulu‐Natal Durban South Africa; ^2^ Faculty of Health Sciences University of the Witwatersrand Durban South Africa; ^3^ Department of Epidemiology Mailman School of Public Health Columbia University New York USA

**Keywords:** comprehensive HIV prevention package, HIV incidence, implementation study, oral pre‐exposure prophylaxis (PrEP), PrEP adherence, PrEP initiation

## Abstract

**Introduction:**

Oral tenofovir disoproxil fumarate/emtricitabine pre‐exposure prophylaxis (PrEP), introduced into South Africa (SA) in 2016, has increasingly become part of HIV prevention standard of care. Given the urgent need for increased HIV prevention efforts for young women in SA, we conducted an implementation study to explore oral PrEP initiation and adherence, and the impact of oral PrEP on HIV incidence in this group.

**Methods:**

This prospective cohort study (CAPRISA 082) was conducted at two sites (urban and rural) in KwaZulu‐Natal, between March 2016 and February 2018. HIV‐negative, sexually active women, aged 18–30 years, were enrolled and followed for approximately 10 months. Oral PrEP was offered as part of a comprehensive HIV prevention package. Adherence to oral PrEP was measured using pill counts and tenofovir‐diphosphate (TFV‐DP) levels. Characteristics of oral PrEP initiators versus non‐initiators were compared using risk ratios. HIV incidence rates were measured using Poisson regression.

**Results:**

Of 425 women enrolled, 262 (62%) initiated oral PrEP. Uptake was significantly higher at the rural site compared to the urban site (78% [*n* = 203/259] vs. 36% [*n* = 59/166], respectively, *p*‐value<0.001). Approximately 25% and 50% had stopped using oral PrEP by 3 and 12 months post‐initiation, respectively. Median pill count adherence was 90% (interquartile range: 81–97%); however, TFV‐DP was only detected in 13% of samples tested, that is 56/431 samples from 97 (37%) participants who initiated oral PrEP. In total, 11 women seroconverted yielding an HIV incidence rate of 2.81 per 100 person‐years (95% confidence interval: 1.40–5.03). Nine of 11 seroconverters had initiated oral PrEP; however, all showed drug levels equivalent to taking one to zero tablets per week. Among women who initiated oral PrEP, >50% had discontinued using oral PrEP by study end, with side effects, such as diarrhoea, nausea, headaches and rash, being the most frequent reason for discontinuation.

**Conclusions:**

Despite moderate oral PrEP initiation and high pill count adherence, adherence as measured by TFV‐DP levels was low and early discontinuation was high. The overall HIV incidence rate was high underscoring the critical need to address barriers to oral PrEP initiation, adherence and continued use, as well as expanding HIV prevention options for young women.

## 1 INTRODUCTION

In 2019, the Joint United Nations Programme on HIV/AIDS estimated that in South Africa (SA), the HIV incidence was 6.9 per 1000 population among adults aged 15–49 years, and of the 190,000 new infections in adults aged ≥15 years, 63% were among women [1]. In sub‐Saharan Africa, five in six new HIV infections among adolescents aged 15–19 years are among girls, and women aged 15–24 years are twice as likely to be living with HIV than men [2]. Factors contributing to this disparity in HIV prevalence among women include poverty, intimate partner violence, intergenerational relationships and biological factors [3, 4]. Heterosexual transmission accounts for majority of HIV infections among women [5]. Increased HIV prevention efforts are needed for women in sub‐Saharan Africa as new HIV infections remain unacceptably high.

In 2015, the World Health Organization recommended that people at substantial risk of HIV infection should be offered oral pre‐exposure prophylaxis (PrEP) as an additional prevention choice, as part of comprehensive prevention [6], and many countries around the world have implemented oral PrEP programmes to varying degrees [7]. Safety and efficacy of tenofovir disoproxil fumarate/emtricitabine (TDF/FTC) as oral PrEP has been demonstrated in several studies [8, 9, 10], with most evidence indicating that oral PrEP works best in men who have sex with men in high‐income settings, and this may be related to biopsychosocial reasons [11]. For young women, oral PrEP may offer additional benefits as it can be initiated without the knowledge or consent of the partner; however, in some instances, taking a bottle of oral PrEP home may result in inadvertent disclosure to partners and/or families and could lead to mistrust, conflict and stigma [12]. Oral PrEP has been approved for use in SA and was initially rolled out by the National Department of Health to select sex worker sites in 2016 [13], but access has since expanded, and oral PrEP is currently available in several demonstration and implementation projects in SA [14].

Generally, adherence to oral PrEP is measured via self‐report, pill counts and where possible measuring tenofovir drug levels. Women who engage in vaginal intercourse need to take oral PrEP daily, without missing any doses, for an optimal HIV prevention effect [15]. However, despite the availability of oral PrEP, initiation, adherence and continuation on oral PrEP has varied in studies among young women in Africa. Earlier studies, such as the FEM‐PrEP and VOICE trials, found low levels of adherence (20–30%) [16, 17]; however, in the Partners PrEP study, tenofovir was detected in 82% of samples [8]. In HPTN082, oral PrEP initiation was high at 95%, and plasma tenofovir (>40 ng/ml) was 48% at 3 months [18]. Other studies/demonstration projects evaluating oral PrEP initiation and adherence among young women have been conducted in several African countries [7], including the EMPOWER [19], POWER [20] and PLUSPILLS [21] studies. Given ongoing high incidence rates of HIV among young women in SA, we undertook this study to determine oral PrEP initiation and adherence, and its impact on HIV incidence rates in young, sexually active women in KwaZulu‐Natal.

## 2 METHODS

### 2.1 Study design

The CAPRISA 082 study was a prospective cohort study conducted at two Clinical Research Sites at urban (eThekwini) and rural (Vulindlela) in KwaZulu‐Natal, SA between March 2016 and February 2018. Volunteers were approached through community outreach activities, participant advocacy, referrals from partner community‐based organizations and/or health facilities, and networking with other recruitment teams within the research site. Sexually active, HIV‐negative women between the ages of 18 and 30 years were enrolled into the study and followed monthly for the first 3 months and seen every 3 months thereafter, for a median of 10 months (interquartile range [IQR]: 7–14). All participants provided written informed consent. The study was approved by the University of KwaZulu‐Natal's Biomedical Research Ethics Committee (BE458/15).

### 2.2 Study procedures

Volunteers were screened for eligibility and eligible participants were enrolled into the study within 30 days of screening. Reimbursement (ranging from ZAR100 to ZAR150) was provided to participants to compensate them for their time, travel and inconvenience.

Rapid HIV antibody finger‐prick testing was performed at each follow‐up study visit. Participants who had positive or discordant HIV rapid tests had blood drawn for HIV confirmatory tests (RNA polymerase chain reaction [PCR], CD3/CD4/CD8^+^ cell counts). Sexually transmitted infections (STIs) (*Chlamydia trachomatis* [CT], *Neisseria gonorrhoea* [NG] and *Trichomonas vaginalis* [TV]) and bacterial vaginosis (BV) testing was performed at enrolment and study exit. STI testing was done using PCR on urine for CT and NG and a genital swab for TV. An HIV risk perception and behavioural assessment using an interviewer‐administered questionnaire was conducted at each visit. Participants were provided with the following HIV prevention options during the study—HIV testing, HIV risk reduction counselling and STI screening, testing and treatment, including the initiation of contact tracing. In addition, women were offered male and female condoms, and oral PrEP. Initiation and, if relevant, adherence to oral PrEP was assessed at each visit and reasons for not initiating or re‐initiating oral PrEP were captured. Blood and genital specimens were collected and stored every 3 months to be used for post‐study assessments of markers of safety, risk exposure, oral PrEP adherence and drug resistance. In addition, stored plasma was used for retrospective RNA PCR testing to confirm whether incident cases of early HIV infection during the study occurred post‐enrolment. Two positive PCR tests at different timepoints confirmed HIV infection. Participants who become infected with HIV were offered counselling and referral, as per SA guidelines [13]. In addition, participants were given the option of being referred to the CAPRISA acute infection study (CAPRISA 002), where immediate antiretroviral therapy initiation was available. Contraceptive services were provided to all participants throughout study participation.

Oral PrEP initiation in CAPRISA 082 was optional and women who chose not to take oral PrEP continued in the study and had access to all services offered within the study as the oral PrEP users. Oral PrEP was contraindicated in individuals with documented HIV infection, creatinine clearance <60 ml/minute and in pregnant or breastfeeding women. The follow‐up procedures that were specific for oral PrEP users were adherence counselling and monitoring, which included pill count and drug‐level monitoring. Given that the drug‐level measurement was not conducted in real time, the results were not used for clinical management. Women who did not initiate oral PrEP or who discontinued oral PrEP were given the option to re‐initiate at any time. During study close‐out, non‐PrEP users were exited from the study first to allow for maximum access/duration on oral PrEP for those who chose to use it. Those who wished to continue oral PrEP were offered participation in a PrEP demonstration project (CAPRISA 084) or were referred to appropriate facilities that offered oral PrEP.

### 2.3 Measures

Oral PrEP initiation was defined as choosing to take oral PrEP daily for HIV prevention. A woman was classified as having discontinued oral PrEP if she reported stopping and not resuming oral PrEP between study visits and did not report re‐initiating oral PrEP again before study closure. Women who did not report stopping but who left the study early (e.g. if they were lost to follow‐up or refused further study participation) were also considered to have discontinued oral PrEP at the date they last reported using oral PrEP.

Adherence to oral PrEP was measured using pharmacy pill count data and using tenofovir‐diphosphate (TFV‐DP) levels in a subset of participants. Pill counts were conducted by a pharmacist at each study visit and the percentage pill count adherence per visit was calculated using the following formula:

$$\frac{No.\;of\;pills\;dispensed\;at\;previous\;visit\;-No.\;of\;pills\;returned/reported\;remaining/lost\;at\;current\;visit}{No.\;of\;pills\;that\;should\;have\;been\;ingested\;between\;visits\;(i.e.\;daily\;pill\;dose\times no.\;of\;days\;between\;visits)}\times 100$$

TFV‐DP levels were retrospectively measured from dried blood spot (DBS) samples from HIV seroconverters and from a subset of participants who had 6 months or more of oral PrEP use and no missed study visits while on oral PrEP. Blood plasma collected from month 3 onwards was used to prepare the DBS samples. A modified liquid chromatography tandem mass spectrometry assay (Division of Clinical Pharmacology, University of Cape Town, SA) was used to measure drug levels [22]. The results were quantified into dosing categories as follows: below lower limit of quantitation, lower limit of quantitation to 349 fmol per punch (fewer than two tablets per week), 350–699 fmol per punch (two or three tablets per week), 700–1249 fmol per punch (four to six tablets per week) and 1250 fmol per punch or more (daily dosing) [23].

Age–disparate relationships were defined as relationships in which a partner is ≥5 years older than the participant. HIV incidence rates were calculated using participant follow‐up time. Date of HIV infection was estimated as the midpoint between the last negative on‐study HIV test date and the first confirmed positive HIV test date (either rapid HIV test or PCR, whichever was earlier). When a participant had a negative rapid test and a positive PCR on the same date, the date of infection was calculated as 14 days prior to this date. Participants who remained HIV uninfected at study end were censored on their study termination date.

### 2.4 Statistical analysis

Data were stratified by rural versus urban. Baseline characteristics of participants were measured using categorical variables and were summarized using frequencies and percentages. The proportion initiating oral PrEP and the proportion who subsequently discontinued oral PrEP was estimated and compared across sites using a Fisher's exact test. The association between participant baseline characteristics and oral PrEP initiation was measured using unadjusted risk ratios with asymptotic 95% confidence intervals (CIs). Time to oral PrEP discontinuation was illustrated using a Kaplan–Meier survival curve. Women who reached the end of study without voluntarily stopping oral PrEP were censored at their study termination date. Oral PrEP adherence was quantified using pill counts and TFV‐DP levels. The frequency and percentage of participants in each TFV‐DP dosing category was estimated overall and by site. Pill count adherence per participant was calculated as the mean of the percentage pill count adherence values from each visit. The median and IQR pill count adherence was calculated using mean pill count adherence values per participant. HIV incidence rates per 100 person‐years (py) and incidence rate ratios were measured using Poisson regression. All statistical analyses were conducted in SAS version 9.4 (SAS Institute Inc., Cary, NC, USA) and used a significance level of 0.05.

## 3 RESULTS

### 3.1 Study cohort population

**Figure 1:**
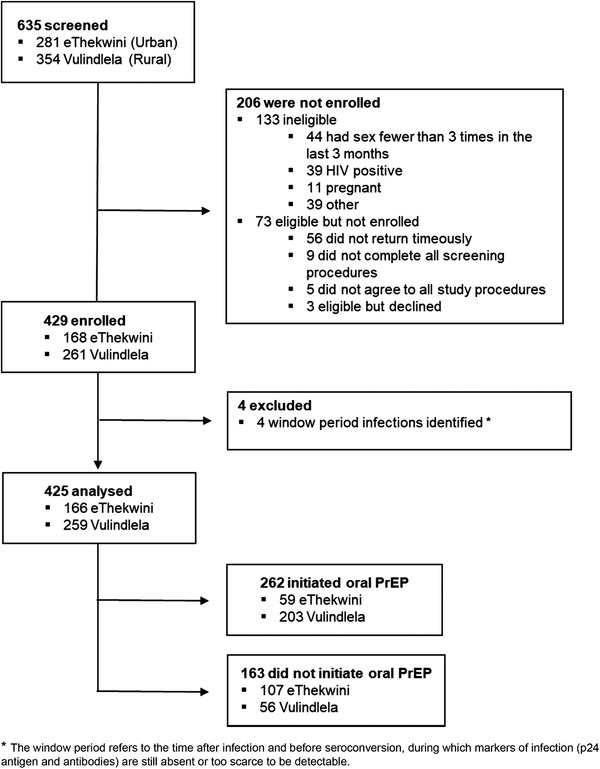
Consort diagram: screening, enrolment and follow‐up of CAPRISA 082 participants.

Of the 635 volunteers screened, 425 (67%) were eligible to participate and enrolled into CAPRISA 082, 166 participants from the urban site (39%) and 259 from the rural site (61%) (Figure 1). Participants were followed‐up for a median of 10 months (IQR: 7–14). Retention rates were 91% and 76% at the urban and rural sites, respectively. At enrolment, approximately two‐thirds of the women were between the ages of 18 and 24 years (67%) (Table 1). Most women enrolled in the study were unemployed (94%) and approximately two‐thirds had completed secondary school or higher (69%). Majority of participants reported having a stable partner (94%) and 22% reported not knowing their partner's HIV status. The prevalence of age–disparate relationships and relationships with migrant workers was 27% and 21%, respectively. BV and one or more STIs were detected in 56% and 22% of participants, respectively.

**Table 1:** Baseline characteristics of CAPRISA 082 participants stratified by site and PrEP initiation

			Urban			Rural		
		Total % (*N*)	Total % (*N*)	Initiated PrEP % (*N*)	RR of PrEP initiation (95% CI)	Total % (*N*)	Initiated PrEP % (*N*)	RR of PrEP initiation (95% CI)
Overall		100 (425)	100 (166)	35.5 (59)	–	100 (259)	78.4 (203)	–
Age	18–24	67.3 (286)	75.9 (126)	35.7 (45)	1.02 (0.63–1.65)	61.8 (160)	75.6 (121)	0.91 (0.81–1.04)
	25–30	32.7 (139)	24.1 (40)	35 (14)	1.00	38.2 (99)	82.8 (82)	1.00
Education	Less than secondary	31.1 (132)	19.3 (32)	31.3 (10)	0.85 (0.49–1.50)	38.6 (100)	79 (79)	1.01 (0.89–1.15)
	Secondary or higher	68.9 (293)	80.7 (134)	36.6 (49)	1.00	61.4 (159)	78 (124)	1.00
Employment	Yes	5.9 (25)	2.5 (4)	0 (0)	–	8.1 (21)	81 (17)	1.04 (0.83–1.29)
	No	94.1 (396)	97.5 (158)	36.7 (58)	–	91.9 (238)	78.2 (186)	1.00
Stable partner	Yes	94.4 (401)	91.6 (152)	34.9 (53)	0.81 (0.43–1.55)	96.1 (249)	78.3 (195)	0.98 (0.71–1.34)
	No	5.6 (24)	8.4 (14)	42.9 (6)	1.00	3.9 (10)	80 (8)	1.00
Married	Yes	1.9 (8)	0.6 (1)	0 (0)	–	2.7 (7)	100 (7)	1.29 (1.20–1.37)^d^
	No	98.1 (417)	99.4 (165)	35.8 (59)	–	97.3 (252)	77.8 (196)	1.00
Casual partner	Yes	5.2 (22)	8.4 (14)	57.1 (8)	1.70 (1.03–2.82)^d^	3.1 (8)	75 (6)	0.96 (0.64–1.43)
	No	94.8 (403)	91.6 (152)	33.6 (51)	1.00	96.9 (251)	78.5 (197)	1.00
Partner age difference	Partner <5 years older or partner younger	73.1 (307)	74.2 (121)	35.5 (43)	1.00	72.4 (186)	80.1 (149)	1.00
	Partner 5–10 years older	23.3 (98)	22.1 (36)	30.6 (11)	0.86 (0.50–1.49)	24.1 (62)	74.2 (46)	0.93 (0.79–1.09)
	Partner >10 years older	3.6 (15)	3.7 (6)	83.3 (5)	2.34 (1.52–3.61)^d^	3.5 (9)	77.8 (7)	0.97 (0.68–1.39)
Partner HIV positive	Yes	2.9 (12)	2.5 (4)	0 (0)	–	3.1 (8)	100 (8)	1.27 (1.18–1.36)^d^
	No	75.3 (317)	80.9 (131)	37.4 (49)	1.00	71.8 (186)	79 (147)	1.00
	Don't know	21.9 (92)	16.7 (27)	37 (10)	0.99 (0.58–1.70)	25.1 (65)	73.8 (48)	0.93 (0.79–1.10)
Partner circumcised	Yes	57.6 (242)	65.2 (105)	38.1 (40)	1.00	52.9 (137)	78.8 (108)	1.00
	No	36.4 (153)	32.3 (52)	30.8 (16)	0.81 (0.50–1.30)	39 (101)	76.2 (77)	0.97 (0.84–1.11)
	Don't know	6 (25)	2.5 (4)	75 (3)	1.97 (1.06–3.65)^d^	8.1 (21)	85.7 (18)	1.09 (0.89–1.32)
Partner migrant worker^a^	Yes	20.6 (64)	4 (5)	40 (2)	1.22 (0.40–3.68)	31.7 (59)	74.6 (44)	0.95 (0.80–1.13)
	No	79.4 (246)	96 (119)	32.8 (39)	1.00	68.3 (127)	78.7 (100)	1.00
Age of sexual debut	<18	38.8 (163)	39.8 (66)	34.8 (23)	0.97 (0.64–1.47)	38.2 (97)	75.3 (73)	0.95 (0.82–1.09)
	> = 18	61.2 (257)	60.2 (100)	36 (36)	1.00	61.8 (157)	79.6 (125)	1.00
Lifetime # sexual partners	1 partner	29.4 (124)	22.9 (38)	23.7 (9)	1.00	33.6 (86)	77.9 (67)	1.00
	2 partners	31.8 (134)	26.5 (44)	34.1 (15)	1.44 (0.71–2.91)	35.2 (90)	75.6 (68)	0.97 (0.82–1.14)
	3+ partners	38.9 (164)	50.6 (84)	41.7 (35)	1.76 (0.94–3.28)	31.3 (80)	83.8 (67)	1.07 (0.93–1.25)
Male/female condom use	Sometimes/always	78 (329)	91.5 (151)	35.1 (53)	0.82 (0.43–1.56)	69.3 (178)	78.1 (139)	0.98 (0.85–1.12)
	Never	22 (93)	8.5 (14)	42.9 (6)	1.00	30.7 (79)	79.7 (63)	1.00
Perceived risk of HIV	No/low risk	42 (178)	53.3 (88)	30.7 (27)	1.00	34.7 (90)	80 (72)	1.00
	Some/high risk	58 (246)	46.7 (77)	40.3 (31)	1.31 (0.87–1.99)	65.3 (169)	77.5 (131)	0.97 (0.85–1.10)
Are you on a non‐barrier contraceptive	Yes	67.5 (286)	66.9 (111)	37.8 (42)	1.22 (0.77–1.94)	67.8 (175)	77.7 (136)	0.98 (0.85–1.12)
	No	32.5 (138)	33.1 (55)	30.9 (17)	1.00	32.2 (83)	79.5 (66)	1.00
Ever used contraceptive pill	Yes	15.8 (67)	17.6 (29)	27.6 (8)	0.75 (0.40–1.41)	14.7 (38)	92.1 (35)	1.21 (1.08–1.36)^d^
	No	84.2 (357)	82.4 (136)	36.8 (50)	1.00	85.3 (221)	76 (168)	1.00
Bacterial vaginosis^b^	Present	56.4 (232)	54.5 (90)	32.2 (29)	0.81 (0.54–1.21)	57.7 (142)	78.9 (112)	1.00 (0.88–1.14)
	Absent	43.6 (179)	45.5 (75)	40.0 (30)	1.00	42.3 (104)	78.9 (82)	1.00
STI detected^c^	Yes	22.4 (93)	16.3 (27)	33.3 (9)	0.93 (0.52–1.65)	26.5 (66)	78.8 (52)	1.01 (0.87–1.17)
	No	77.6 (322)	83.7 (139)	36 (50)	1.00	73.5 (183)	78.1 (143)	1.00
Time of PrEP initiation	At enrolment	84.0 (220)	55.9 (33)			92.1 (187)		
	After enrolment	16.0 (42)	44.1 (26)			7.9 (16)		

^a^ Migrant worker is a person who moves to another country or area in order to find employment. One hundred and fifteen participants did not respond to this question.

^b^ Fourteen results bacterial vaginosis tests not done.

^c^ STIs comprised trichomoniasis, chlamydia and gonorrhoea. Ten missing STI results.

^d^ *p*‐value<0.05.

### 3.2 Oral PrEP initiation

Overall, oral PrEP initiation was 62% (*n* = 262/425). Women at the urban site were less likely than those at the rural site to initiate oral PrEP (36% [*n* = 59/166] vs. 78% [*n* = 203/259], *p*‐value<0.001) and more likely to delay initiation (44% vs. 8%, *p*‐value<0.001) (Table 1). Among women at the urban site, oral PrEP initiation was positively associated with being >10 years younger than their partner (relative risk [RR] (95% CI): 1.70 [1.03–2.82]) compared to those with a partner <5 years older than them, and with having a casual partner (RR [95% CI]: 2.34 [1.52–3.61]) (Table 1). At the rural site, married women (RR [95% CI]: 1.29 [1.20–1.37]), women reporting HIV‐positive partners (RR [95% CI]: 1.27 [1.18–1.36]) and women who previously or currently used the oral contraceptive pill (RR [95% CI]: 1.21 [1.08–1.36]) were more likely to initiate oral PrEP. Frequent reasons cited by participants during the study for not initiating or re‐initiating oral PrEP include lack of interest (39%), a perception of being unlikely to adhere to daily dosing (34%) and a dislike for taking tablets (33%) (Table 2).

**Table 2:** Reasons for CAPRISA 082 participants not initiating PrEP during follow‐up, stratified by site

Reason^a^	All sites		Urban		Rural	
	(*N* = 163)		(*N* = 107)		(*N* = 56)	
Not interested/willing to take PrEP	63	39%	9	8%	54	96%
Likely to forget to take pills	56	34%	54	50%	2	4%
Do not like taking tablets	53	33%	47	44%	6	11%
Not ready to initiate yet	49	30%	46	43%	3	5%
Concerned about adherence	30	18%	30	28%	0	0%
Concerned about side effects	28	17%	25	23%	3	5%
Self‐perceived no HIV risk	20	12%	10	9%	10	18%
Other reason not initiated	18	11%	14	13%	4	7%
Ineligible for PrEP^b^	17	10%	5	5%	12	21%
Breastfeeding	15	9%	0	0%	15	27%
Concerned about objection family	10	6%	10	9%	0	0%
Risk of developing resistance	2	1%	2	2%	0	0%

^a^ Participants were asked to provide reasons at each visit that they did not initiate PrEP and could cite more than one reason for not initiating PrEP.

^b^ Included participants who were awaiting confirmatory HIV tests, those using traditional medicine and those with deranged LFT.

### 3.3 Oral PrEP discontinuation

**Figure 2:**
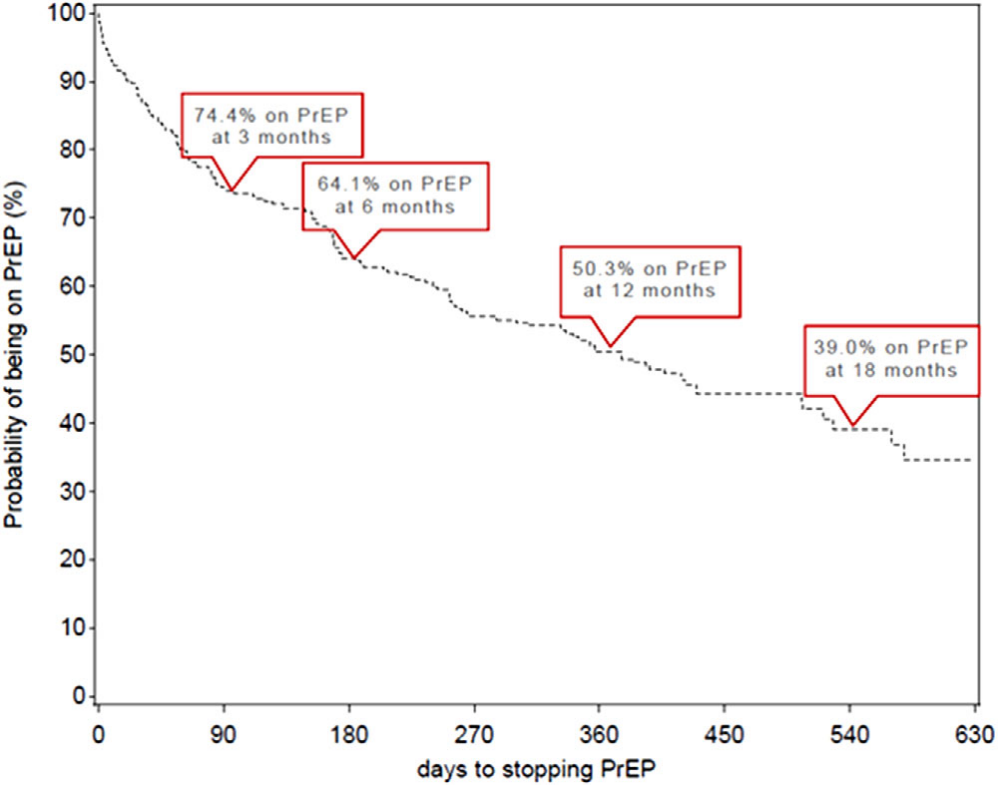
Time (days) to stopping PrEP use among CAPRISA 082 participants who initiated PrEP.

Of the 262 women initiated on oral PrEP, 67 (26%) and 94 (36%) had discontinued oral PrEP within 3 and 6 months of initiation, respectively. The probability of staying on oral PrEP for longer than 12 months was 50.3% (Figure 2). By the end of the study, 145 (55%) of the 262 initiated on oral PrEP had discontinued their oral PrEP. The proportion that discontinued oral PrEP before early study closure was higher in the urban site than the rural site (64% [*n* = 38/59] vs. 52% [*n* = 105/203], *p*‐value = 0.1024). The most common reasons given for stopping oral PrEP provided by participants who discontinued oral PrEP but continued in the study (*n* = 101/143) were side effects (27%). Approximately 65% of participants who initiated oral PrEP reported side effects. The most common self‐reported side effects were diarrhoea (21%), nausea (18%), headaches (18%) and rash (17%), with the highest proportion of side effects (37%) being reported within 1 month of oral PrEP initiation.

### 3.4 Oral PrEP adherence

**Figure 3:**
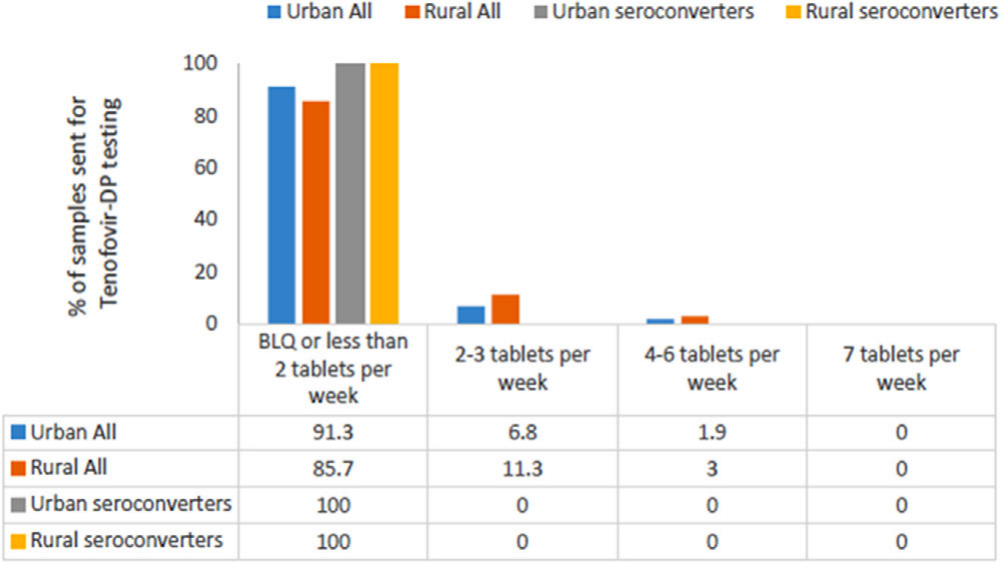
Adherence to PrEP measured by tenofovir‐DP levels seen among a subset of CAPRISA 082 participants.

TFV‐DP levels were measured in 431 DBS samples from 97 (37%) of the 262 participants who initiated oral PrEP, with an average of four samples tested per participant. Detected drug levels were low, with 91% (94/103) and 86% (281/328) of samples from the urban and rural sites, respectively, measuring below the lower limit of quantification or less than 349 fmol per punch (two tablets per week) (Figure 3). Pill count adherence was, however, high. Overall median (IQR) pill count adherence was 90% (81–97%) and for those participants with drug levels was 97% (89–98%).

### 3.5 HIV incidence

In total, 11 participants seroconverted during 391 py of follow‐up, resulting in an HIV incidence rate of 2.81 per 100 py (95% CI: 1.40–5.03) overall, and an HIV incidence rate of 3.95 per 100 py (95% CI: 1.28–9.22) and 2.26 per 100 py (95% CI: 0.83–4.93) at the urban and rural sites, respectively (Table S1). The HIV incidence rate was 3.19 per 100 py (95% CI: 1.70–5.58) among those who initiated oral PrEP and 1.84 per 100 py (95% CI: 0.57–5.11) among those who did not. Nine of the 11 seroconverters had initiated oral PrEP during the study. However, of the 25 samples collected from the nine seroconverters, all showed drug levels that were below 349 fmol per punch (equivalent to ≥1 tablet per week) (Figure 3). Median (IQR) pill count adherence for these nine seroconverters at the visits at which drug levels were measured was 94% (91–99%). Antiretroviral drug resistance testing was done on all seroconverters, and no clinically significant resistance mutations were detected.

## 4 DISCUSSION

We observed a moderate initiation of oral PrEP among young women aged 18–30 years in our cohort, with almost two‐thirds initiating oral PrEP overall, and a significantly higher initiation at the rural site. More than half of the women felt that they were at some/high risk of HIV acquisition. One in five women had an STI detected at enrolment. While adherence to oral PrEP as measured by pill counts was high, only 13% of the samples had TFV‐DP levels that were above or equal to 350 fmol/punch. More than half of those who initiated oral PrEP had discontinued using oral PrEP before study end, and the most frequent reason for discontinuation was side effects. Overall HIV incidence was 2.81 per 100 py and 3.19 per 100 py among oral PrEP users.

A high initiation of oral PrEP has been observed among young African women aged 16–25 years in several recent studies, for example in the HPTN082 and POWER studies, oral PrEP initiation was >90% [18, 20]. However, in clinical trials where oral PrEP was provided as part of HIV prevention standard of care, initiation has been lower—in the ECHO trial, where oral PrEP was provided on‐site during the latter part of the trial, initiation of oral PrEP was 27% at the SA trial sites [24], while in the HVTN702 vaccine study, self‐reported oral PrEP use was <1% [25]. In our study, initiation of oral PrEP at the rural site was more than double the urban site (78% vs. 36%). A greater proportion of women at the urban site reported choosing not to initiate oral PrEP for the following reasons—likely to forget taking pills, did not like taking pills, concerns about side effects, not feeling ready to initiate oral PrEP and concerns about adherence, compared to the rural site, where most women who did not initiate oral PrEP reported they were not interested or not willing to take oral PrEP. One of the early lessons that emerged from oral PrEP implementation projects among adolescent girls and young women in Africa was the importance of oral PrEP awareness and demand creation with positive messaging about the benefits of oral PrEP [21, 26]. In other studies, reasons for declining oral PrEP have included low perceived risk of acquiring HIV, having an HIV uninfected partner, pill burden and adherence concerns [27, 28].

Among women who initiated oral PrEP in our study, adherence as measured by pill counts was high; however, <15% of the samples had TFV‐DP levels that were more than or equal to 350 fmol/punch. Similarly, in both the FEM‐PrEP and VOICE trials among African women, while pill counts suggested high levels of adherence, measured tenofovir was only detected in 15–30% of samples [16, 17]. This indicates that the pill count was an unreliable adherence measurement as participants may be altering the number of pills they bring back to the clinic due to the “white coat effect.” In HPTN082, despite the high initiation of oral PrEP, detectable TFV‐DP declined from 84% at 3 months, to 31% at 1 year [18]. Reasons for non‐adherence to oral PrEP can vary, and a systematic review of adherence to oral PrEP which included studies from Africa found that reasons for non‐adherence over multiple studies were stigma, low risk perception, low decision‐making power, an unacceptable dosing regimen, side effects and the logistics of daily life [29]. It is also possible that biomedical methods, such as pills for HIV prevention, are new to this setting/population and time may be needed to “trust” new HIV technologies, noting that the oral PrEP rollout in SA only commenced in 2016 and was initially limited to select sex worker sites [13]. In addition, further advertising, promotional materials and most importantly provider information and training may be needed. Oral PrEP drop‐off rates increased over time, with just over 10% discontinuing at month 1, a quarter at month 3 and a third at month 6. However, by the end of the study, more than half the women had discontinued using oral PrEP, and side effects were cited as the most frequent reason for discontinuation. This drop off was also observed among adolescents in the PLUSPILLS study [21], and in women offered oral PrEP in the ECHO Trial at a single study site, almost 40% of those who discontinued oral PrEP had done so due to side effects [30].

The HIV incidence rates among oral PrEP users in our study (3.19 [95% CI: 1.70–5.58] per 100 py) were high but comparable to that of other studies. In the POWER study, HIV incidence was 2.1 per 100 py [20] and in HPTN082, 1.0 per 100 py [18]. Similar high HIV incidence rates were observed in the VOICE and FEM‐PrEP trials (5.7 per 100 py and 4.7 per 100 py, respectively) [16, 17]. More recently, the ECHO trial also demonstrated a high HIV incidence of 3.8 per 100 py [24], and HVTN702 demonstrated an HIV incidence of >4 per 100 py among women [25]. The high HIV incidence observed in our study and several others among women in Africa demonstrates the urgent need for effective HIV prevention methods in this setting. It is not surprising that there was no statistically significant difference in HIV incidence among participants in our cohort who initiated oral PrEP versus those who did not, as none of the seroconverters had detectable TFV‐DP. This highlights the need for enhanced adherence counselling among this population, as well as less user‐dependent PrEP options for young women in Africa who are unable or unwilling to take daily oral PrEP.

Our study has a few limitations. In practice, monetary compensation will not be provided to individuals accessing oral PrEP from public healthcare facilities; however, in our study, accessing services and oral PrEP was made easier by reimbursement. Although this reimbursement was offered regardless of oral PrEP initiation, women who may otherwise not have initiated oral PrEP because of transport or other costs associated with going to their local clinic, may have decided to initiate oral PrEP at the research site. This, in turn, may have contributed to the lack of correlates of oral PrEP initiation and the high discontinuation observed in this study. Our study was conducted soon after the approval of oral PrEP for use in SA, and oral PrEP access and availability was very limited during this time (2016–2018). More recently, oral PrEP access and availability has increased in SA, and this could influence oral PrEP initiation, adherence and continued use. Our findings should be supplemented with outcomes from demonstration projects and implementation science research conducted post‐2018 to effectively impact on policy/programmes in SA.

## 5 CONCLUSIONS

Despite a moderate initiation of oral PrEP in our study, pill count adherence was high, but tenofovir drug levels were poor, with more than half of the women who initiated oral PrEP discontinuing use within 12 months. Notwithstanding, HIV incidence rate remains high. There is a critical need to identify and address barriers to oral PrEP initiation, adherence and continued use. Other less user‐dependent HIV prevention options for young women in Africa, who are unable or unwilling to take daily oral PrEP, are urgently required.

## COMPETING INTERESTS

All authors have no competing interests.

## AUTHORS’ CONTRIBUTIONS

LEM: conception and design, study management, data collection and manuscript writing; LL: technical advice for statistical aspects, data analysis and manuscript writing; CLN: data analysis and manuscript writing; IH, HD and KN: study coordination and data collection, TNG: provided technical advice for pharmacy aspects, PrEP supply chain management, assay interpretation guidance and data collection. NS: provided technical advice for laboratory aspects and data collection. IB: manuscript writing; SSAK and QAK: conception, design and critical revision of the manuscript. All authors read and approved the final manuscript.

## DISCLAIMER

The content of this publication does not necessarily reflect the views, analysis or policies of FHI 360, USAID or the United States Government, nor does any mention of trade names, commercial products or organizations imply endorsement by these organizations.

## Supporting information


**Supplementary Table 1**: HIV incidence rates and incidence rate ratios by participant characteristic.

## Data Availability

The datasets used and/or analysed during the current study are available from the corresponding author on reasonable request.
