# Supplementary material for: Prospective study of oral pre‐exposure prophylaxis initiation and adherence among young women in KwaZulu‐Natal, South Africa
Source: J Int AIDS Soc. 2022 Jul 3;25(7):e25957. doi: 10.1002/jia2.25957 (PMC9251857; doi:10.1002/jia2.25957)
Supplement: Supplementary file 1 — Supplementary Table 1: HIV incidence rates and incidence rate ratios by participant characteristic. [file JIA2-25-e25957-s001.docx]

**Supplementary Table 1: HIV incidence rates and incidence rate ratios by participant characteristic**

|  |  | No. seroconversions/no. person-years | Incidence per 100 person-years (95% CI) | Incidence rate ratio (95%CI) |
| --- | --- | --- | --- | --- |
| Overall |  | 11/391 | 2.81(1.40-5.03) | - |
| Site | Urban | 5/127 | 3.95(1.28-9.22) | 1.74(0.53-5.72) |
|  | Rural | 6/265 | 2.26(0.83-4.93) | 1.00 |
| PrEP Initiation | No | 2/109 | 1.84(0.22-6.63) | 0.58(0.12-2.67) |
|  | Yes | 9/283 | 3.18(1.46-6.04) | 1.00 |
| Age | 18-24 | 7/256 | 2.74(1.1-5.64) | 0.93(0.27-3.18) |
|  | 25-30 | 4/136 | 2.94(0.8-7.54) | 1.00 |
| Education | Less than secondary | 3/123 | 2.43(0.5-7.11) | 0.82(0.22-3.07) |
|  | Secondary or higher | 8/268 | 2.98(1.29-5.88) | 1.00 |
| Partner Age Difference | Partner >5 years older | 2/81 | 2.46(0.3-8.9) | 0.84(0.18-3.88) |
|  | Partner <5 years older, or partner younger | 9/306 | 2.94(1.34-5.57) | 1.00 |
| Partner HIV Positive | Yes | 1/12 | 8.03(0.2-44.73) | 3.33(0.41-27.03) |
|  | Don't know | 3/87 | 3.45(0.71-10.09) | 1.43(0.37-5.53) |
|  | No | 7/290 | 2.41(0.97-4.97) | 1.00 |
| Partner Circumcised | No | 6/144 | 4.17(1.53-9.07) | 2.25(0.64-7.98) |
|  | Don't know | 1/28 | 3.53(0.09-19.65) | 1.91(0.21-17.06) |
|  | Yes | 4/216 | 1.85(0.5-4.74) | 1.00 |
| Partner Migrant Worker* | Yes | 2/61 | 3.27(0.4-11.8) | 1.08(0.22-5.19) |
|  | No | 7/231 | 3.03(1.22-6.24) | 1.00 |
| Lifetime # Sexual Partners | 1 partner | 4/116 | 3.45(0.94-8.85) | 1.00 |
|  | 2 partners | 1/126 | 0.79(0.02-4.41) | 0.23(0.03-2.05) |
|  | 3+ partners | 6/147 | 4.07(1.49-8.86) | 1.18(0.33-4.17) |
| Male/Female Condom Use | Sometimes/always | 9/301 | 2.99(1.37-5.68) | 1.00 |
|  | Never | 2/89 | 2.25(0.27-8.13) | 0.75(0.16-3.48) |
| Worried About HIV | Not worried at all | 1/49 | 2.06(0.05-11.47) | 1.00 |
|  | Worried to some extent | 10/343 | 2.92(1.4-5.37) | 1.42(0.18-11.08) |
| Perceived Risk of HIV | No/low risk | 4/156 | 2.57(0.7-6.57) | 1.00 |
|  | Some/high risk | 7/235 | 2.98(1.2-6.13) | 1.16(0.34-3.96) |
| Bacterial Vaginosis | Present | 7/217 | 3.23(1.3-6.65) | 1.3(0.38-4.45) |
|  | Absent | 4/161 | 2.48(0.68-6.35) | 1.00 |
| STI Detected** | Yes | 2/86 | 2.33(0.28-8.42) | 0.77(0.17-3.54) |
|  | No | 9/296 | 3.04(1.39-5.78) | 1.00 |

*Migrant worker is a person who moves to another country or area in order to find employment.

**STIs comprised trichomoniasis, chlamydia and gonorrhoea

^^^ p-value<0.05
